# More Fair Play in an Ultimatum Game after Resettlement in Zimbabwe: A Field Experiment and a Structural Model

**DOI:** 10.1371/journal.pone.0064791

**Published:** 2013-05-28

**Authors:** Stefan Kohler

**Affiliations:** Institute for Social Medicine, Epidemiology and Health Economics, Charité University Medical Center, Berlin, Germany; Hungarian Academy of Sciences, Hungary

## Abstract

Zimbabwean villagers of distinct background have resettled in government-organized land reforms for more than three decades. Against this backdrop, I assess the level of social cohesion in some of the newly established communities by estimating the average preferences for fairness in a structural model of bounded rationality. The estimations are based on behavioral data from an ultimatum game field experiment played by 234 randomly selected households in 6 traditional and 14 resettled villages almost two decades after resettlement. Equal or higher degrees of fairness are estimated in all resettlement schemes. In one, or arguably two, out of three distinct resettlement schemes studied, the resettled villagers exhibit significantly higher degrees of fairness (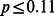 ) and rationality (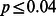 ) than those who live in traditional villages. Overall, villagers appear similarly rational, but the attitude toward fairness is significantly stronger in resettled communities (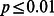 ). These findings are consistent with the idea of an increased need for cooperation required in recommencement.

## Introduction

The government of Zimbabwe has implemented land reform schemes to address inequalities in land ownership since gaining independence in 1980. Numerous Zimbabwean households have resettled in government-organized land reform programs in the past, and several resettled people faced the challenge of restarting along with unfamiliar people. According to *Wu et al.* [1], studies show that social distance influences people's justice concerns [2]–[5] and people's other-regarding behavior [6]–[8]. A field experiment in Southern Ethiopia found higher levels of generosity towards known family members than towards anonymous villagers [9]. The Zimbabwean land reform, therefore, poses the question whether villagers in new communities lost social capital and, in the long-run, suffer from an eradication of social ties compared to villagers who did not resettle. I address this question by studying the following subquestions: Does social cohesion exist among former strangers after resettlement in some instances? If so, how much? And, how to reliably measure social cohesion or an interpretable factor of it? To answer these questions, I evaluate if different social preferences are present in some of the communities of the early Zimbabwean land reform period and their non-resettled counterparts almost two decades after once unfamiliar households became neighbors through resettling.

I measure social cohesion roughly by estimating the relative strengths of self-interest in relation to an interest in fairness, defined as an aversion toward inequality between one's own and another's well-being. As I have no comparable information on the fairness preferences of the villagers before resettlement, the evidence on preference differences put forward in this study does not allow to infer what was causing them. The estimated differences in the villagers preferences might either be the result of resettlement or have been preexisting to resettlement and, thus, reflect a self-selection of more fair-minded individuals into the land reform program. Notwithstanding the initial conditions, some of which are documented by *Dekker*, *Deininger et al.* or *Kinsey* [10]–[12], one possible outcome of resettlement could be an erosion of trust [13] or other factors of social cohesion through the increased social distance among resettled villagers compared to traditional villagers. The study at hand suggests that resettlement did not eradicate social cohesion in resettled villages.

I explore the levels of social cohesion in three resettlement schemes (Mupfurudzi, Mutanda, Sengezi) of the first period of Zimbabwean land reform and in geographically close traditional communities. Data stem from an ultimatum game field experiment preceding this study [13] and were also part of a large-scale study of cross-cultural variation in behavior [14], [15]. The choice problem given in the ultimatum game was to be solved anonymously by randomly matched pairs of villagers within 14 resettled and 6 traditional communities. The decision task in the ultimatum game experiment mimics the common feature of bargaining situations that different parties have to reach an agreement to realize a mutually beneficial outcome. The approach of applying a structural decision model to this field experimental data in order to disentangle self-interested, fairness and rationality as competing explanations for the variations in experimental behavior that were observed across regions and resettlement status is novel. It has two distinct advantages over the analysis of the descriptive statistics of the game: Firstly, out-of-sample predictions become possible as with any estimated model. Secondly, the equilibrium model applied allows the extrapolation of the parameters estimated to other settings than the experiment because the estimates have a defined interpretation that is meaningful also outside the experimental context.

A quantal response equilibrium is applied to estimate the relative strength of fairness in relation to self-interest. It is a game-theoretic solution concept, which combines an equilibrium notion that accounts for strategic optimizing behavior with bounded rationality [16], [17]. Players are assumed to make random errors in choosing which pure strategy to play, but the probability of any particular strategy being chosen increases in its payoff such that more costly errors are less likely. The randomness introduced transforms the deterministic model in a stochastic model that allows for maximum-likelihood estimation of the model's parameters.

The estimation results indicate significant degrees of fairness and bounded rationality in addition to self-interest for all of the three different areas studied. All resettled villagers (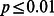 ), resettled villagers in the Sengezi area (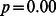 ) and arguably resettled villagers in the Mutanda area (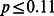 ) show significantly higher degrees of fairness than their traditional village counterparts. The pooled data of all areas do not reject the hypothesis that resettled and traditional villagers are, on average, equally rational. The assumption of common rationality for the Mutanda and Sengezi areas is rejected (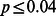 ) and significantly higher degrees of rationality are estimated for their resettled villagers. A higher degree of fairness and a lower degree of rationality are estimated for resettled villagers in Mupfurudzi, but their difference from the preferences and rationality of traditional villagers is insignificant. The positive correlation between fairness and rationality in the Sengezi and Mutanda areas is consistent with the idea that social cohesion accompanies villagers' experience in cooperative interaction with unrelated households that, presumably, occurred more frequently in resettled villages.

This study makes also a methodological contribution. As for the disaggregated data none of the differences that are estimated significant in the equilibrium model are significant in the descriptive statistics of the ultimatum game behavior and vise versa, my study provides an example for how an equilibrium assessment of the ultimatum game changes the level of analysis compared to a non-equilibrium approach.

### Land Reform in Zimbabwe

The land policy of the Government of Zimbabwe can be divided into two periods: A *willing-buyer and willing-seller* land reform program existed for two decades from independence to 2000. Donors assisted the Zimbabwean government to finance the purchase of land, and the land reform resettled over 70,000 indigenous households on farms previously owned by white commercial farmers until 1997. The scheme targeted individual households of displaced people, the landless and those with insufficient land to sustain themselves and their families. The beneficiaries were allocated five hectares of arable land for cultivation in a resettlement site and the remaining area was devoted to communal grazing land. Households were also allocated a residential plot within newly planned villages. As the majority of households resettled on an individual basis, resettled villagers, unlike traditional villagers, started to live largely among unrelated households instead of their kin [10], [11], [18], [19]. “To qualify for land reform, several categories of potential beneficiaries were distinguished. Eligible for settlement were: (a) refugees and other persons displaced by war; (b) those residing in communal areas who were landless; and (c) those who had insufficient land to maintain themselves and their families. In addition, to be eligible, household heads were supposed to be married or widowed, aged 25–55 and not in formal employment. Broadly speaking, these criteria seem to have been followed. Some 90% of households settled in the early 1980s had been adversely affected by the war for independence in some form or another. Before being resettled, most (66%) had been peasant farmers with most of the remainder being landless laborers on commercial farms, workers in the rural informal sector or wage earners in the urban sector” [11]. “But whatever the reason for resettlement, it is safe to assume that there were many more households in similar situations who did not resettle, either because their applications were unsuccessful or because they found traditional means of accessing land and dealing with personal trauma more attractive” [13].

Studying the benefits and the costs of the *willing-buyer and willing-seller* land reform program in Zimbabwe, a positive but modest economic return, between 5 and 8 percent, on the investment in land reform was estimated [11]. According to *Deininger et al.* the assessment of the performance of the land reform program depends largely on the perspective taken: Taking a household perspective, some research indicates that resettled villagers managed to accumulate large amounts of (livestock) assets or to increase their productivity tremendously [20], [21]. In terms of per capita performance criteria, such as per capita expenditure or nutritional status, traditional villagers outperformed resettled villagers [22], [23].

A *fast-track* land reform that ended the *willing-buyer and willing-seller* land reform was begun by President Mugabe in 2000. It seized white-owned farms and its legality and constitutionality have been challenged in the Zimbabwean High and Supreme Courts. On the one hand, the *fast-track* land reform has been criticized for its socioeconomic consequences: “Mr. Mugabe's chaotic land redistribution campaign [...] caused an exodus of white farmers, crippled the economy, and ushered in widespread shortages of basic commodities” [24]. One the other hand, it is argued that a new rural economy has developed ten years after large areas of Zimbabwe's commercial farm land were compulsorily transferred without compensation: “A wide range of activities contribute to highly differentiated livelihoods in [some of] the new resettlements” [25]. In sum, the *fast-track* and *willing-buyer-willing-seller* land reforms took place in different macroeconomic and political contexts with distinct resettlement practices.

### Related Literature

I review two related strands of literature. Firstly, I discuss studies which argue that social preferences models predict observed behavior accurately and studies which investigated heterogeneity in social preferences in distinct sociocultural contexts. These previous studies motivated my estimation of social preferences in the two distinct environments created by the Zimbabwean *willing-buyer and willing-seller* land reform. Secondly, I review studies that analyzed the very same data before and discuss what this study adds. Background information on the early period of Zimbabwean land reform or attempts to evaluate it are not reviewed, but provided, for instance, by *Dekker*, *Deininger et al.* or *Kinsey* [10]–[12].

Previous studies suggests that models of social preferences reproduce observed behavior consistently when estimated in a quantal response equilibrium of different experimental games: Bounded rationality and significant degrees of altruism reproduce behavior in public goods games [26], [27]. Linear or quadratic fairness and decision error predict the patterns of positive offers and rejections in, as well as across, bargaining games [28], [29]. Hence, social preferences, which capture forms of (conditional) altruism in addition to self-interest, might be an appropriate model to explain behavior across games. However, how well can diversity in the strength of social preferences explain behavior across individuals and societies? *Bellemare et al.* used data from ultimatum and dictator games played online by a large representative Dutch population sample to estimate nonlinear preferences for equity combined with limited rationality. Heterogeneous equality preferences together with subjective expectations predict their observed decisions well [30]. *Barr et al.* investigate to what extent behavioral variations observed in three bargaining games could be attributed to differences along a single dimension, namely the value placed on equality. The bargaining games were played in 15 distinct societies, ranging from US undergraduates to Amazonian, Arctic, and African hunter-gatherers. Testing a number of predictions implied by a utility function, which captures the same notion of quadratic inequality aversion as employed in this study, they conclude that “inequality aversion is the principle motivating factor [in all societies] and variations in behavior across societies and across individuals within societies do, in large part, result from differences in the value placed upon equality” [31]. Since equality preferences seem to capture the behavior in bargaining games well, even across sociocultural contexts, these studies motivated my choice to estimate a model of quadratic inequality aversion based on ultimatum game bargaining data in order to measure social cohesion among the sampled Zimbabwean villagers in the study at hand.

The same methodology and Zimbabwean ultimatum game data were used previously. Estimating social preferences on the study population level, *Kohler* finds no evidence for gender-related differences in the Zimbabwean villagers' preferences, a result that could be due to the small sample size. However, he finds that resettlement status significantly impacts the value villagers place on equality. This finding was robust in a limited test of the model specification, which suggested that a model of symmetric inequality aversion fits the aggregate ultimatum game data better than a model of aversion merely to disadvantageous situations. The earlier study also compared estimates from ultimatum games played in small-scale societies and industrialized countries, and it argues that higher levels of decision error are estimated for small-scale societies even when correcting for the different purchasing power of the money at stake in the games [32]. In the present analyses, I employ *Kohler*'s model specification with symmetric inequality aversion that fitted the data better to trace the difference in social preferences observed after resettlement on the regional level.

The data, which I reanalyze on a more disaggregate level, has been collected by *Barr* who first evaluated the ultimatum game (UG) data and additional trust game data to make a behavioral comparison between the two groups of villages. She found that game behavior was consistent with, firstly, there being no differences in socially transmitted behavioral rules and, secondly, the hypothesis of similar altruism or loyalty towards co-villagers in both village groups. Thirdly, *Barr* describes her data as consistent with the hypothesis that resettled villagers behave more cautiously when in strategic situations with their co-villagers [13]. Due to optimizing behavior, in the form of strategic anticipation of the other's incentives, and the abstract experimental context, it is, however, in question how to interpret the differences detected in the descriptive statistics of the UG behavior and how to learn from these differences beyond the context of the game? Higher proposals in the UG may not only result from social cohesion or fairness considerations. In particular, also greater uncertainty about the responder's rejection behavior may cause self-interested precautionary high proposals. In the study at hand, I resolve these issues by linking the behavior that is observed in the UG to underlying incentives and the degree of rationality of the experiment participants, which are then compared, rather than observed action. Moreover, the quantal response equilibrium model of social preferences can be adapted to other game-theoretic problems. Thus, unlike the UG raw data, it can be used to obtain a plausible forecast of behavior in other contexts than the UG, after its parameters are estimated.

## Materials and Methods

### Data

Data were generated by *Barr* who conducted an UG field experiment in Zimbabwe in 1999 [13]. Her sample covers 234 households, i.e., 117 matched pairs of UG bargainers, in 14 newly established villages and six traditional communities nearby geographically. The sampled villages were all among the first resettled villages created in 1982, within three resettlement schemes. The Mupfurudzi and Sengezi resettlement schemes are each compared to the two traditional villages in the adjacent areas that provided most settlers to the resettled villages in the sample (see [10], [19]). The households from which the players in each of the subject pools originated were participants in a long-term monitoring exercise. It was designed to assess the effects of resettlement in Zimbabwe. The samples of subjects for the experiments matched the sample of households in the monitoring exercise to the extent possible. In some villages both UGs and trust games were played. In other villages only one game selected at random was played [13]. The geographical location of the research sites is mapped in *Dekker* or *Deininger et al.* [10], [11].

Resettlement villages differed from traditional villages in a number of ways around the time of the field experiment (Table 1). Household size, the proportions of aged people, livestock wealth, and within-village kinship ties varied significantly between resettled and traditional households (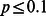 ). Some of these variables, along with other variables, varied significantly between resettled and traditional villages, even within the three areas [19]. Each resettlement scheme's area represents one of Zimbabwe's three agriculturally most important agroclimatic zones that correspond to regions of moderately high, moderate and restricted agricultural potential: Mupfurudzi in Mashonaland Central Province, Mutanda in Manicaland Province and Sengezi in Mashonaland East Province [18]. While the traditional villages exist since the 1940s and 1950s, the inhabitants' ancestors lived together before, giving the social structure a longer tradition that differs from the resettled communities [10], [33]. Within villages, participants for the UG experiment were recruited by inviting each household to send a member above the age of 14. The headman was asked to oversee that between forty and sixty percent of the volunteers were women. Play in the games was anonymous, non-recurring and for stakes between a half and two day's local casual labor wage. The stakes in the administered UG were Zim$ 50 and the smallest unit to be offered was Zim$ 5. Since the subject pool was small and experimentees knew they were playing with someone from their village, observed play was seen to be likely to reflect experiences from the day-to-day communal interaction [13]. The proportion of households sampled in the experiments varied between 0.31 and 0.59 or was 1. Average earnings were between half a day's and a day's casual wage. The exchange rate at the time of the experiment was Zim$ 37.95 per US$ 1.

**Table 1:** Comparison of household demographics, wealth, within-village linkages, and village ethnic composition in resettled and traditional areas.

	Resettled villages		Traditional villages		Range of village means	
In the years 1999 to 2001	N	MN	N	MN	Resettled	Traditional
Nonreligious memberships	557	4.09	143	1.52^*^	1.00–7.50	0.78–3.14
Household size	394	9.39	143	5.9^*^	5.73–14.20	5.00–6.88
Women 15-60 y	394	0.28	143	0.27	0.20–0.34	0.20–0.32
Young <15 y	394	0.4	143	0.41	0.33–0.50	0.42–0.46
Aged >60 y	394	0.08	143	0.11^*^	0.03–0.12	0.06–0.20
Livestock	568	13.59	145	7.71^*^	7.29–25.15	6.35–7.47
Marriage ties	753	0.81	188	0.91	0.06–1.87	0.53–1.86
Extended family ties	753	5.76	188	0.55^*^	0.00–1.56	1.42–10.81
Nuclear family ties	753	0.3	188	1.91^*^	0.00–0.68	0.82–2.83
Some initial social capital	723	0.83	245	1^*^	0.42–1.00	1.00–1.00
Ethnic dominance	22	40.77	6	49.67	0.19–1.00	0.32–0.70
Ethnic diversity	22	5.95	6	5.33	1.00–11.00	4.00–6.00
Number of households	22	37	6	46.17^*^	13.00–64.00	34.00–63.00

Adopted from *Barr* who provides the variable definitions and assumes that close and extended family ties predate resettlement and so can count as a special type of initial condition. Some initial social capital reflects the proportion of households that knew any other households prior to resettlement who resettled nearby; it is set equal to 1 for traditional villages. Data sources: *Barr, Dekker and Kinsey* [10], [13], [40].

The UG itself is a strategic situation, in which two players are allotted a sum of money (the stakes) and then bargain about its division. The first player, called the proposer, makes an offer, which the second player, the responder, can accept or reject. If the responder accepts then the stakes are divided according to the proposed split. If the responder rejects, both receive nothing. Subgame perfect equilibrium and own money maximization predict that proposers should offer the smallest non-zero amount and responders should always accept because they face a choice between zero and something. In contrast, as observed in numerous prior UG experiments (e.g. [34]), the behavior within the UG experiments in all Zimbabwean villages substantially deviated from narrow self-interest: Firstly, offers are positive, averaging 38 to 48 percent of the stakes, and the unique mode for all but Mupfurudzi's traditional proposers is fair division. Secondly, offers below 30 percent of the stakes were rejected by 33 to 57 percent of responders in all but Sengezi's traditional villages, in which 17 percent of the responders rejects such low offers (Table 2, rows 1–4).

**Table 2:** Summary of ultimatum game bargaining experiment.

		All			Mupfurudzi			Mutanda			Sengezi		
Villagers	Action	N	MN	SD	N	MN	SD	N	MN	SD	N	MN	SD
All	Offer	117	0.44	0.11^a^	34	0.44	0.11^b^	19	0.45	0.10	64	0.44	0.12
	Rejection rate	117	0.08	0.27	34	0.06	0.24	19	0.05	0.23	64	0.09	0.29
	… if offer<0.5	38	0.24	0.43^c^	12	0.17	0.39	5	0.20	0.45	21	0.29	0.46
	… if offer<0.3	13	0.46	0.52^d^	3	0.33	0.58^e^	2	0.50	0.71	8	0.50	0.54
Resettled	Offer	86	0.45	0.10	25	0.46	0.08	9	0.48	0.04	52	0.45	0.12
	Rejection rate	86	0.07	0.26	25	0.04	0.20	9	0.00	0.00	52	0.10	0.30
	… if offer<0.5	24	0.25	0.44	7	0.14	0.38	2	0.00	0.00	15	0.33	0.49
	… if offer<0.3	7	0.57	0.54	1	1.00		0			6	0.50	0.55
Traditional	Offer	31	0.41	0.14	9	0.38	0.17	10	0.43	0.13	12	0.41	0.13
	Rejection rate	31	0.10	0.30	9	0.11	0.33	10	0.10	0.32	12	0.08	0.29
	… if offer<0.5	14	0.21	0.43	5	0.20	0.45	3	0.33	0.58	6	0.17	0.41
	… if offer<0.3	6	0.33	0.52	2	0.00	0.00	2	0.50	0.71	2	0.50	0.71

Superscripts denote significant differences in the mean UG behaviors of resettled and traditional villagers in the area. ^a^T-test and Mann-Whitney Wilcoxon test for the equality of mean offers (*p*≤0.05). ^b^T-test for the equality of mean offers (*p*≤0.10). ^c^Chi-square test for the equality of the rejected proportions of offers smaller than half (*p*≤0.10). ^d^Fisher's exact test for the equality of the rejected proportions of offers smaller than 30 percent (*p*≤0.10). ^e^Fisher's exact test for the equality of the rejected proportions of offers smaller than 30 percent (*p* **= **0.16). Data source: *Barr* [13].

Comparing the behavior of traditional and resettled villagers (Table 2, rows 5–12) shows that mean offers in villages of the three resettlement schemes are between 45 and 48 percent compared to 38 to 43 percent in traditional communities nearby geographically. While all average offers are larger in resettled villages, a two-sided *t*-test indicates that the difference is significant only in the pooled and in the Sengezi sample at the 5 and 10 percent level, respectively. A Mann-Whitney Wilcoxon test further indicates a difference in the overall offer distribution at the 5 percent level. Chi-square and Fisher's exact tests show no statistical evidence for difference in the overall rejection rate of the offers observed, but for differences in the average rejection rate of low offers below 50 and 30 percent in the pooled sample as well as for the rejection rate of offers below 30 percent in Sengezi at the 10 and 16 percent level, respectively. An equal partition of the money at stake is proposed most frequently in 69 and 55 percent of all play in resettled and traditional villages, respectively. The most generous offer of 60 percent of the stakes is observed three times in two resettled villages in the Mupfurudzi area.

### Model

Positive average offers and rejection of small proposals in the UG resemble typical experimental behavior that has been studied extensively. This behavioral pattern is predicted, for instance, by social preferences which assume that players care about fairness in addition to self-interest [7], [11], [25]. Such social preferences trigger positive offers in the UG through inequality averse enough responders *R*, who reject small offers. Independently of the triggered positive offers, non-zero offers will also be made by proposers *P* who are themselves substantially inequality averse.

Adopting the functional representation of concern for fairness proposed by *Bolton and Ockenfels* [7], I estimate the coefficient of a quadratic loss function by which deviations from payoff equity diminish the utility gained from one's own payoff:
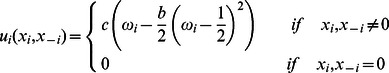



Surplus 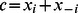 is the sum of individual payoffs and 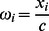 is the proportion of the surplus that the player 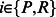 gets. Parameter *b* measures the importance of relative gains in comparison to one's own monetary payoff. It is interpreted as fairness and assumed common to a population. For 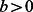 , all things being equal, utility decreases if there is an inequality in payoffs, regardless whether it is to a player's advantage or disadvantage. This effect is the stronger, the larger an inequality is. In the UG, such utility implies that the responder rejects unequal proposals if 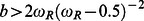 . The proposer, intrinsically or together with anticipation of a fair-minded responder's rejection of small or large offers, may offer higher amounts than implied by pure self-interest. As the degree of fairness *b* co-determines behavior in the subgame perfect equilibrium of the UG, variations therein give a rationale for the varying degree of sharing behavior observed between resettled and traditional villagers in the Zimbabwean UG field experiment.

An anticipated random shock shall add to the expected utility and cause players to not always choose their best strategy given their beliefs in order to capture variation in the optimal play of individuals who share one and the same preferences. Each player knows that the other player does so as well and anticipates the preferences and decision error of the other. As a consequence, all strategies of a player can be observed with some probability. The equilibrium for a given error structure is defined as a fixed point of this process with mutual correct anticipation. *McKelvey and Palfrey* established the existence of this quantal response equilibrium (QRE) under the assumptions that players maximize utility and estimate expected payoffs in an unbiased way [16], [17]. The original QRE is based on a game's normal-form, which disregards that, in the UG, the responder can make his choice between accepting and rejecting the offer after observing the offer [16]. The QRE was extended to extensive-form games later by proposing an agent QRE. The agent QRE is defined similarly to the normal-form QRE, but for the agent normal-form of an extensive-form game, in which different information sets of a given player are assumed to be played independently by different agents who share the same payoff function [17]. This study applies the agent QRE.

In the UG played in the Zimbabwean villages, the proposer *P* chooses a strategy 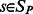 in his discrete strategy set 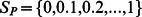 of eleven possible offers, in which the stakes of Zim$ 50 are normalized to one. The portion *s* denotes how much the proposer offers to the responder *R* and 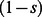 is what he would like to keep for himself. The behavioral strategy 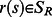 of the responder is a function that maps each possible offer into his dichotomous strategy set 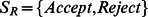 . Assuming the best response functions follow a logit distribution, the resulting QRE is often called a logit equilibrium, in which the players' optimal mixed strategies are determined by the pair of probabilities that solve:
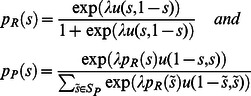



The numerators are exponential functions of the expected payoffs that result from the UG behavior. The denominators are normalizing factors that force the probabilities to sum to one. The distribution parameter 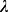 is a common measure of rationality, which implies that each strategy is chosen with equal probability if 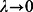 and that the expected utility maximizing strategy is chosen with certainty if 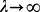 . As surplus *c* and the degree of rationality 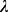 are interchangeable in the logit response functions 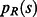 and 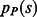, the size of surplus affects the estimated degree of rationality. All estimates reported for 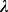 are based on 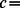 Zim$ 50.

The logit response functions 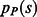 and 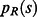 also define the logit equilibrium for the UG. They imply that strategies with higher expected payoffs are chosen with higher probability. The logit equilibrium approaches a subset of Nash equilibria as rationality increases, i.e. decision errors decrease [16], [38]. Since the random utility shocks in the model cause differently experienced utility at identical behavior, one interpretation of the QRE framework is that it represents heterogeneity between individuals, which is not covered by the preference parameter *b*. The intuition of QRE solution and its application to the UG in more detail is discussed elsewhere (e.g. [32]).

### Estimation

The stochastic equilibrium prediction of the logit equilibrium, in contrast to the deterministic Nash equilibrium, allows to obtain maximum likelihood estimates of the strength of fairness *b* relative to self-interest and the degree of rationality 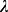 . As the joint density of 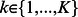 independent and identically distributed observations is given by multiplying the probabilities to observe each individual outcome, the log-likelihood of observing a particular sample of *k* observations in the UG is given by:
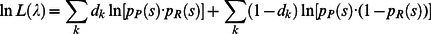



Dummy 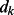 assumes unity for acceptance and naught for rejection of *s*. The identification of parameters *b* and 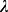 in the likelihood function is warranted through the functional form of the employed utility function. The degree of rationality 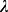 is affected proportionally and inequality aversion *b* disproportionately high by payoff variations.

## Results and Discussion

### Model Fit

**Figure 1:**
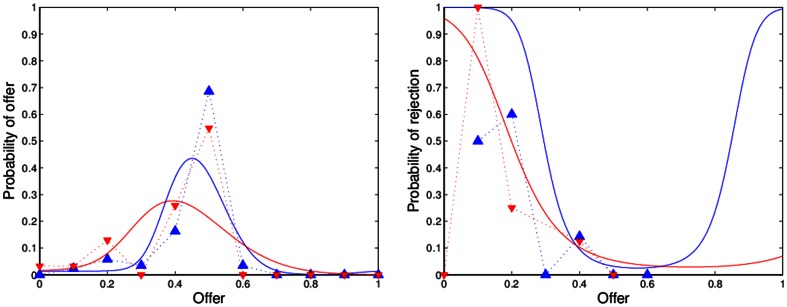
Observed and predicted offer distributions and rejection rates in all areas. Dashed lines indicate actual play. Solid lines indicate model prediction. Blue lines and upward-pointing triangle indicate resettled villages. Red lines and downward-pointing triangle indicate traditional villages. Data source: *Barr* [13].

**Figure 2:**
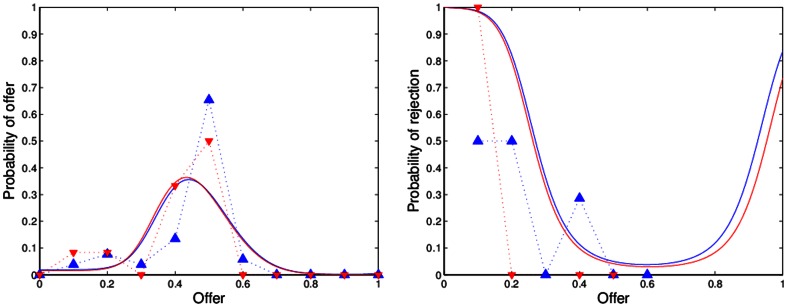
Observed and predicted offer distributions and rejection rates in Mupfurudzi. Dashed lines indicate actual play. Solid lines indicate model prediction. Blue lines and upward-pointing triangle indicate resettled villages. Red lines and downward-pointing triangle indicate traditional villages. Data source: *Barr* [13].

**Figure 3:**
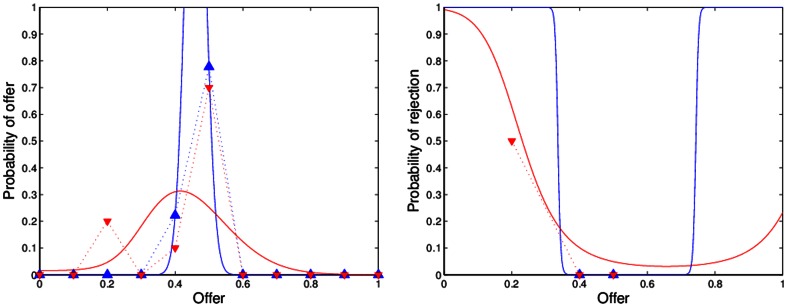
Observed and predicted offer distributions and rejection rates in Mutanda. Dashed lines indicate actual play. Solid lines indicate model prediction. Blue lines and upward-pointing triangle indicate resettled villages. Red lines and downward-pointing triangle indicate traditional villages. Data source: *Barr* [13].

**Figure 4:**
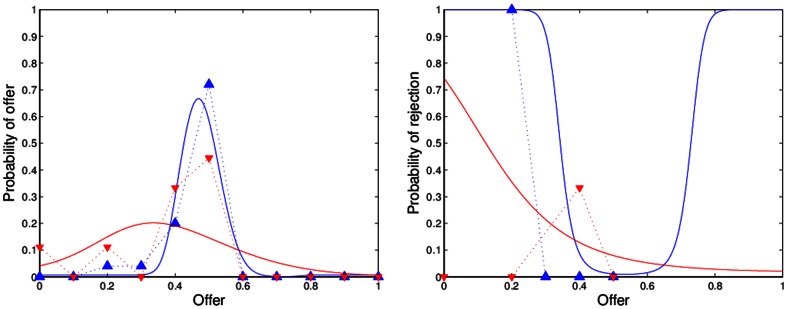
Observed and predicted offer distributions and rejection rates in Sengezi. Dashed lines indicate actual play. Solid lines indicate model prediction. Blue lines and upward-pointing triangle indicate resettled villages. Red lines and downward-pointing triangle indicate traditional villages. Data source: *Barr* [13].

Figures 1, 2, 3, 4 show the observed and predicted play in resettled and traditional villages, overall and for each sample area. All curves of the predicted behavior (solid lines) show that the model resembles the observed high frequency (dashed lines) of mid-range offers along with higher rejection rates of low offers for the resettled villagers and universal decline in rejection rates for offers smaller than half. Higher degrees of rationality are reflected by a smaller spread of the offer distribution. Pronounced interest in fairness causes an incline in the forecast rejection rate for offers smaller or larger than half, but offers larger than 60 percent are not observed. Table 3 presents the distribution of observed and predicted offers by area and resettlement status. The corresponding Table 4 summarizes the actual and forecast rejection rates. The model with one and the same estimates for resettled and traditional villagers is reported for the Mupfurudzi area as it will later not be rejected by the data.

**Table 3:** Observed and predicted offer distributions.

	All						Mupfurudzi			Mutanda						Sengezi				
	b_r_≠b_t_ ∧ λ_r_≠λ_t_						b_r_ = b_t_ ∧ λ_r_ = λ_t_			b_r_≠b_t_ ∧ λ_r_≠λ_t_						b_r_≠b_t_ ∧ λ_r_≠λ_t_				
	Resettled			Traditional			All			Resettled			Traditional			Resettled			Traditional	
Offer	N	Actual	Model	N	Actual	Model	N	Actual	Model	N	Actual	Model	N	Actual	Model	N	Actual	Model	N	Actual
0	0	0	0.01	1	0.03	0.01	0	0	0.02	0	0	0.00	0	0	0.00	0	0	0.01	1	0.11
0.1	2	0.02	0.01	1	0.03	0.01	3	0.05	0.02	0	0	0.00	0	0	0.00	0	0	0.01	0	0
0.2	5	0.06	0.01	4	0.13	0.01	5	0.08	0.03	0	0	0.00	2	0.20	0.00	1	0.04	0.01	1	0.11
0.3	3	0.03	0.06	0	0	0.06	2	0.03	0.12	0	0	0.00	0	0	0.00	1	0.04	0.01	0	0
0.4	14	0.16	0.36	8	0.26	0.36	11	0.17	0.33	2	0.22	0.22	1	0.10	0.22	5	0.20	0.30	3	0.33
0.5	59	0.69	0.37	17	0.55	0.37	40	0.63	0.31	7	0.78	0.78	7	0.70	0.78	18	0.72	0.58	4	0.44
0.6	3	0.03	0.12	0	0	0.12	3	0.05	0.13	0	0	0.00	0	0	0.00	0	0	0.07	0	0
0.7	0	0	0.02	0	0	0.02	0	0	0.03	0	0	0.00	0	0	0.00	0	0	0.00	0	0
0.8	0	0	0.00	0	0	0.00	0	0	0.00	0	0	0.00	0	0	0.00	0	0	0.01	0	0
0.9	0	0	0.00	0	0	0.00	0	0	0.00	0	0	0.00	0	0	0.00	0	0	0.01	0	0
1	0	0	0.01	0	0	0.01	0	0	0.00	0	0	0.00	0	0	0.00	0	0	0.01	0	0
Correlation^P^	0.73^***^			0.78^***^			0.68^***^			1.00^***^			0.96^***^			0.97^***^			0.98^***^	

*b_r_*≠*b_t_* ∧ *λ_r_*≠*λ_t_* indicates the offer distributions are predictions of a model with unequal fairness and rationality estimates that depend on resettlement status; *t* traditional, *r* resettled. *b_r_* **=** *b_t_* ∧ *λ_r_* **=** *λ_t_* indicates predictions of a model with similar fairness and rationality estimates for resettled and traditional villagers.

P Weighted correlation between observed and predicted proposer behavior, i.e., offers. Stars denote significance at 1 percent level. Data source: *Barr* [13].

**Table 4:** Observed and predicted rejection rates.

	All						Mupfurudzi			Mutanda						Sengezi				
	b_r_≠b_t_ ∧ λ_r_≠λ_t_						b_r_ = b_t_ ∧ λ_r_ = λ_t_			b_r_≠b_t_ ∧ λ_r_≠λ_t_						b_r_≠b_t_ ∧ λ_r_≠λ_t_				
	Resettled			Traditional			All			Resettled			Traditional			Resettled			Traditional	
Offer	N	Actual	Model	N	Actual	Model	N	Actual	Model	N	Actual	Model	N	Actual	Model	N	Actual	Model	N	Actual
0	0		1.00	1	0	0.96	0		1.00	0		1.00	0		0.99	0		1.00	1	0
0.1	2	0.50	1.00	1	1	0.81	3	0.67	0.99	0		1.00	0		0.92	0		1.00	0	
0.2	5	0.60	0.95	4	0.25	0.50	5	0.40	0.82	0		1.00	2	0.50	0.63	1	1	1.00	1	0
0.3	3	0	0.47	0		0.23	2	0	0.36	0		1.00	0		0.26	1	0	0.91	0	
0.4	14	0.14	0.09	8	0.13	0.10	11	0.18	0.11	2	0	0.00	1	0	0.10	5	0	0.09	3	0.33
0.5	59	0	0.03	17	0	0.05	40	0	0.05	7	0	0.00	7	0	0.05	18	0	0.01	4	0
0.6	3	0	0.03	0		0.03	3	0	0.04	0		0.00	0		0.03	0		0.02	0	
0.7	0		0.05	0		0.03	0		0.05	0		0.00	0		0.03	0		0.22	0	
0.8	0		0.24	0		0.03	0		0.11	0		1.00	0		0.05	0		0.98	0	
0.9	0		0.82	0		0.04	0		0.36	0		1.00	0		0.09	0		1.00	0	
1	0		0.99	0		0.07	0		0.82	0		1.00	0		0.23	0		1.00	0	
Correlation^R^	0.91^***^			0.64^***^			0.91^***^			NA			1.00^***^			0.72^***^			–0.24	
Correlation^PR^	0.73^***^			0.69^***^			0.65^***^			1.00^***^			0.97^***^			0.89^***^			0.45^*^	

*b_r_*≠*b_t_* ∧ *λ_r_*≠*λ_t_* indicates the offer distributions are predictions of a model with unequal fairness and rationality estimates that depend on resettlement status; *t* traditional, *r* resettled. *b_r_* **=** *b_t_* ∧ *λ_r_* **=** *λ_t_* indicates predictions of a model with similar fairness and rationality estimates for resettled and traditional villagers.

R Weighted correlation between observed and predicted responder behavior, i.e., rejection rates. *^PR^*Weighted correlation between observed and predicted proposer and responder behavior, i.e., offers and rejection rates. Stars denote significance at the 10 and 1 percent level. Data source: *Barr* [13].

The estimated model correctly predicts the unique mode of equal division in the UG in all areas but Mupfurudzi and substantial low offer rejection. The forecast and actual proposer behavior for all data result in weighted correlation coefficients of 0.73 (resettled) and 0.78 (traditional); the forecast and actual responder behavior result in correlation coefficients of 0.91 (resettled) and 0.64 (traditional). Comparing the decisions of proposers and responders in Mupfurudzi, Mutanda and Sengezi with predicted behavior generally indicates a good fit of the model estimated by area. The forecast and actual responder behavior result in significant correlation coefficients of 0.65 to 1 in all areas but Sengezi. For traditional Sengezi responders the model over-predicts the low offer rejection probability of the two rejected lowest offers and fails to capture its peak of 33 percent rejections at offers of 40 percent, leading to an insignificant negative correlation of –0.24 between observation and prediction. However, a single rejection of the two lowest offers instead of certain acceptance in the traditional Sengezi communities would result in a significant positive correlation between actual and forecast behavior. If the negative correlation was true, it could point to a failure in the model's structure. The model that imposes one and the same preferences on proposers and responders may, for instance, not represent the decision-making of responders in Sengezi. Due to the few available observations this assumption is not tested, but I argue for its plausibility because villagers were assigned to a role in the UG randomly. If the UG itself does not affect the inert preferences, then there is no reason to expect systematic differences between proposers and responders. In fact, the model fits the other data well. Hence, imposing the same preference structure for proposers and responders in Sengezi can be a safeguard against data mining in a single subsample.

### Parameter Estimates

The model is fitted to the data in the quantal response equilibrium of the sequential UG assuming the villagers' choices were mutual best responses, given their own and anticipating the other's preferences and degree of rationality. The estimated degree of fairness measures the extent to which observed offers and rejections are explained by fairness felt toward others, taking into account the occurrence of decision error and rejection of low offers. Based on having assumed an appropriate model structure, the estimated model parameters, therefore, reflect the extent to which rejections were made because of a violation of an intrinsic fairness norm or because of decision errors. The estimated decision error is part of the model and does not reflect the model fit of the data.

Table 5 summarizes the parameter estimates of three different model specifications by resettlement status. The full sample consists of 117 pairs of observations. The estimation results are firstly presented for the full sample (Table 5, rows 1–3) and then by area (Table 5, rows 4–12). The table reports the estimation results for the unrestricted Model 1, in which fairness and rationality may vary across the resettlement status; the restricted Model 2, in which solely rationality may vary across the resettlement status; and the restricted Model 3, in which solely fairness may vary across resettlement status. A restricted model (not reported), in which rationality may vary across the resettlement status, whereas pure self-interest is assumed, is universally rejected in favor of a reported model by likelihood-ratio tests. Standard errors are bootstrapped with 1000 repetitions. The p-values (not reported) corresponding to models reported in Table 5 indicate that the estimates of each model, but two traditional Sengezi specifications, are significant at the 1 percent level. This confirms the presumption that decision errors and fairness are both necessary to explain the variation in play well in the majority of cases. No interest in fairness may prevail in traditional Sengezi villages if fairness was estimated only within this subsample (Table 5, Models 1 and 3, row 12; 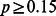 ).

**Table 5:** Estimation results of the logit equilibrium models.

			Model 1					Model 2					Model 3				
			b_r_≠b_t_ ∧ λ_r_≠λ_t_					b_r_ = b_t_ ∧ λ_r_≠λ_t_					b_r_≠b_t_ ∧ λ_r_ = λ_t_				
			b		λ			b		λ			b		λ		
Area	Villagers	N	Coef.	SE	Coef.	SE	–ln L	Coef.	SE	Coef.	SE	–ln L	Coef.	SE	Coef.	SE	–ln L
All	All	117	9.80	3.76	6.19	0.55	210.07										
All	Resettled	86	13.97	6.12	6.83	0.74	139.30	10.38	4.17	6.81	0.71	140.52	14.04	6.15	6.55	0.56	139.44
	Traditional	31	4.39	6.03	5.74	2.62	64.68			4.86	3.31	67.34	3.80	6.39			65.09
Mupfurudzi	All	64	10.04	5.39	5.96	0.64	118.00										
Mutanda	All	19	17.82	16.19	7.15	13.23	28.84										
Sengezi	All	34	8.18	8.62	6.35	4.84	61.79										
Mupfurudzi	Resettled	52	10.21	6.91	5.89	0.71	96.37	10.05	5.61	5.90	0.73	96.37	10.19	7.02	5.96	0.66	96.37
	Traditional	12	9.30	13.43	6.28	17.91	21.59			6.26	10.81	21.60	9.41	14.67			21.62
Mutanda	Resettled	9	25.09	16.73	49.03	15.08	4.77	22.19	12.44	100.00	40.36	4.79	37.58	22.97	8.45	15.20	6.47
	Traditional	10	6.39	29.71	5.93	16.30	18.86			5.37	24.40	20.10	3.87	29.69			19.69
Sengezi	Resettled	25	27.86	11.09	9.02	17.89	28.66	14.58	11.33	9.07	27.74	29.89	26.77	12.48	7.40	4.22	29.44
	Traditional	9	1.74	11.03^a^	4.90	12.10	22.23			1.85	10.33	25.99	1.09	11.92^b^			23.62

*N* denotes the number of proposer and responder pairs. *b_r_*≠*b_t_* ∧ *λ_r_*≠*λ_t_* denotes a model with fairness and rationality parameters that can depend on resettlement status; *t* traditional, *r* resettled. *b_r_* **=** *b_t_* ∧ *λ_r_*≠*λ_t_* denotes a model with similar fairness, but different rationality parameters for resettled and traditional villag ers. *b_r_*≠*b_t_* ∧ *λ_r_* **=** *λ_t_* denotes a model with different fairness, but similar rationality parameters for resettled and traditional villagers. ln *L* denotes the log-likelihood of the fitted model. Standard errors are bootstrapped with 1000 repetitions. Coefficients are significant at the 1 percent level unless marked otherwise. The upper bound of *λ*≤100 was imposed as an upper bound in the maximum likelihood estimation. ^a,b^Bootstrapped p-values are 0.15 and 0.20, respectively. Data source: *Barr* [13].

Model 1 shows that, overall, positive degrees of fairness and rationality are estimated (Table 5, row 1). Splitting the sample according to resettlement status, I find a significantly higher degree of fairness in resettled than in traditional villages (13.97 vs. 4.39). At the same time the degree of rationality is of similar magnitude (6.83 vs. 5.74) without significant difference such that, overall, a model of different fairness (14.04 vs. 3.80), but similar rationality (6.55) is accepted by the data (Table 5, rows 2–3). Table 6 reports the corresponding likelihood-ratio tests that reject the equality of both coefficients between resettled and traditional villagers, jointly and individually (Table 6, rows 1–3; 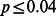 ), but not an equal degree of rationality after different degrees of fairness were estimated (Table 6, row 5; 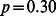 ). By contrast, an equal degree of fairness is rejected after different degrees of rationality were estimated (Table 6, row 4; 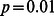 ).

**Table 6:** Summary of hypotheses and tests.

	Hypothesis		Number of parameters			–ln L			
Area	H_0_	H_1_	H_0_	H_1_	df	H_0_	H_1_	LR	P-value
All	b_r_ ** = **b_t_ ∧ λ_r_ ** = **λ_t_	b_r_≠b_t_ ∧ λ_r_≠λ_t_	2	4	2	210.07	203.99	12.16	0.00
	b_r_ ** = **b_t_ ∧ λ_r_ ** = **λ_t_	b_r_ ** = **b_t_ ∧ λ_r_≠λ_t_	2	3	1	210.07	207.87	4.40	0.04
	b_r_ ** = **b_t_ ∧ λ_r_ ** = **λ_t_	b_r_≠b_t_ ∧ λ_r_ ** = **λ_t_	2	3	1	210.07	204.53	11.07	0.00
	b_r_ ** = **b_t_ ∧ λ_r_≠λ_t_	b_r_≠b_t_ ∧ λ_r_≠λ_t_	3	4	1	207.87	203.99	7.76	0.01
	b_r_≠b_t_ ∧ λ_r_ ** = **λ_t_	b_r_≠b_t_ ∧ λ_r_≠λ_t_	3	4	1	204.53	203.99	1.09	0.30
Mupfurudzi	b_r_ ** = **b_t_ ∧ λ_r_ ** = **λ_t_	b_r_≠b_t_ ∧ λ_r_≠λ_t_	2	4	2	118.00	117.96	0.09	0.96
	b_r_ ** = **b_t_ ∧ λ_r_ ** = **λ_t_	b_r_ ** = **b_t_ ∧ λ_r_≠λ_t_	2	3	1	118.00	117.98	0.06	0.81
	b_r_ ** = **b_t_ ∧ λ_r_ ** = **λ_t_	b_r_≠b_t_ ∧ λ_r_ ** = **λ_t_	2	3	1	118.00	117.99	0.02	0.88
	b_r_ ** = **b_t_ ∧ λ_r_≠λ_t_	b_r_≠b_t_ ∧ λ_r_≠λ_t_	3	4	1	117.98	117.96	0.03	0.86
	b_r_≠b_t_ ∧ λ_r_ ** = **λ_t_	b_r_≠b_t_ ∧ λ_r_≠λ_t_	3	4	1	117.99	117.96	0.07	0.80
Mut.	b_r_ ** = **b_t_ ∧ λ_r_ ** = **λ_t_	b_r_≠b_t_ ∧ λ_r_≠λ_t_	2	4	2	28.84	23.62	10.43	0.01
	b_r_ ** = **b_t_ ∧ λ_r_ ** = **λ_t_	b_r_ ** = **b_t_ ∧ λ_r_≠λ_t_	2	3	1	28.84	24.88	7.90	0.00
	b_r_ ** = **b_t_ ∧ λ_r_ ** = **λ_t_	b_r_≠b_t_ ∧ λ_r_ ** = **λ_t_	2	3	1	28.84	26.16	5.36	0.02
	b_r_ ** = **b_t_ ∧ λ_r_≠λ_t_	b_r_≠b_t_ ∧ λ_r_≠λ_t_	3	4	1	24.88	23.62	2.52	0.11
	b_r_≠b_t_ ∧ λ_r_ ** = **λ_t_	b_r_≠b_t_ ∧ λ_r_≠λ_t_	3	4	1	26.16	23.62	5.07	0.02
Sengezi	b_r_ ** = **b_t_ ∧ λ_r_ ** = **λ_t_	b_r_≠b_t_ ∧ λ_r_≠λ_t_	2	4	2	61.79	50.89	21.79	0.00
	b_r_ ** = **b_t_ ∧ λ_r_ ** = **λ_t_	b_r_ ** = **b_t_ ∧ λ_r_≠λ_t_	2	3	1	61.79	55.87	11.83	0.00
	b_r_ ** = **b_t_ ∧ λ_r_ ** = **λ_t_	b_r_≠b_t_ ∧ λ_r_ ** = **λ_t_	2	3	1	61.79	53.06	17.46	0.00
	b_r_ ** = **b_t_ ∧ λ_r_≠λ_t_	b_r_≠b_t_ ∧ λ_r_≠λ_t_	3	4	1	55.87	50.89	9.97	0.00
	b_r_≠b_t_ ∧ λ_r_ ** = **λ_t_	b_r_≠b_t_ ∧ λ_r_≠λ_t_	3	4	1	53.06	50.89	4.34	0.04

*df* denotes degrees of freedom. ln *L* denotes the log-likelihood of the fitted model. *LR* denotes the likelihood-ratio test statistic. P-values stem from a Chi-squared distribution. *r,t* indicate that estimate depends on resettlement status. Data source: *Barr* [13].

Repeating this assessment for each of the three sampled resettlement schemes of Mupfurudzi, Mutanda and Sengezi, I do not detect statistical evidence for different fairness or rationality between resettled and traditional Mupfurudzi villagers (Table 6, rows 6–10; 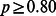 ). Equality of both coefficients between resettled and traditional villagers, jointly and individually, is rejected in the Mutanda and Sengezi areas (Table 6, rows 11–13 and 16–18; 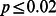 ). Detecting different preferences in Mutanda, for which descriptive statistics did not indicate behavioral differences, provides a salient example of the different level of analysis that is imposed by the structural equilibrium model compared to non-equilibrium analyses of the UG data. For the Sengezi area, further likelihood-ratio tests reject that variation in a single parameter is sufficient to explain the observed differences (Table 6, rows 19–20; 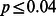 ). For the Mutanda area, I reject only various degrees of fairness (Table 6, row 15; 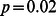 ). I also tentatively reject only differences in the villagers' degree of rationality with an 11 percent probability of error (Table 6, row 14; 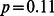 ). As the Mutanda area is the smallest subsample in the analyses with only 19 pairs of observations, the likelihood-ratio test applied could lack power to depict the relatively large difference in estimated fairness (Table 5, Model 1, rows 9–10; 25.09 vs. 6.39) as significant. The nine pairs of resettled Mutanda villagers' play resulted in only two outcomes involving seven equal partitions and two 40 percent offers, all of which were accepted. The low degree of bounded rationality needed to explain these deviations causes their degree of rationality to take the highest values in the sample. For the restricted model of unique fairness, the rationality estimate 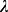 equals its upper bound 100.

In the Mutanda and Sengezi resettlement schemes, the higher degree of fairness occurs jointly with a higher degree of rationality. This finding supports the idea that resettled villagers not only had an increased need of cooperativeness after their recommencement but also gained experience in cooperating in the past, which, in turn, is facilitated by their higher fairness preferences. When the model is estimated with all data, the data of villagers from three different resettlement schemes are pooled. The resulting estimates indicate a higher degree of fairness for resettled villagers, but a similar degree of rationality for all villagers. That is consistent with the intuition that the decision error estimates which capture the heterogeneity between individuals not covered by the social preference parameter converge in a larger sample, whereas the increased need for cooperation in recommencement remains an experience shared only by resettled villagers.

### Limitations

The findings discussed are subject to limitations. Preferences were not estimated on the individual, but on the area and resettlement status level. Decision error is the way the model accounts for heterogeneity between individuals. The assumed structural form of the model was only tested against one alternative, an asymmetric specification of inequality aversion (see [32]). The model remains motivated by its success to predict this and other behavioral data. The analyses are based on a small data sample from an UG experiment in the field with 117 pairs of observations and little variation in the observed behavior. Sixty-five percent of the proposing players offer half, 92 percent of the responding players accept. These limiting factors are exacerbated within the three regional subgroup analyses of the impact of resettlement on the preferences. In spite of these limitations, a coherent picture emerged: Average fairness is estimated higher for resettled than for traditional villages in each area. The difference is significant in Sengezi and arguably Mutanda, but insignificant in the Mupfurudzi area. Significantly higher fairness estimates are accompanied by significantly lower decision errors, potentially reflecting more experience with cooperation amongst the resettled villagers. None of the findings should be causally attributed to resettlement without further information because the villagers' choice to resettle may have been correlated with their fairness attitudes and rationality. If the more (less) fair-minded self-selected themselves in the resettlement program, then the reported fairness estimates would overestimate (underestimate) the effect of resettlement on equality preferences. Even though voluntary participation in the resettlement program may seem to suggest that non self-interested motives were at work, the formal eligibility criteria of being a refugee or displaced, landless or without sufficient land are also compatible with a purely self-interested motivation to volunteer for the resettlement program which allocated land to its beneficiaries. I do not account for either form of selection into the resettlement program.

## Conclusions

I reexamined preexisting experimental data from ultimatum games conducted in the aftermath of the Zimbabwean 1980s *willing-buyer and willing-seller* land reform. A structural model attributed observed ultimatum game behavior to its potential origins. Based on the experimental behavior of 234 resettled and traditional villagers, I estimated social preferences on the community level that incorporate a utility loss when the bargaining villagers obtain other than a fair division of the money at stake in the game. The strength of the utility loss in comparison to self-interest was interpreted as concern for fairness, the assumed proxy for social cohesion. The quantal response equilibrium model used to estimate villagers' average concern for fairness introduced a notion of bounded rationality that assumes players make similar decision errors, which are negatively related to the payoff from that decision. Decision error could be interpreted as an estimate of heterogeneity in fairness between individuals. In the equilibrium, the players were assumed to maximize utility and correctly anticipate subsequent behavior. The estimates of fairness and decision error were obtained by maximizing the likelihood of observing the experimental behavior.

Significantly positive degrees of fairness and bounded rationality forecast bargaining behavior accurately for most participants of the experiments in resettled and traditional villages of the Mupfurudzi, Mutanda and Sengezi areas. The correlations of observed and predicted behavior are between 0.45 and 1. In the supraregional estimations of the model, the aggregate data do not reject the hypothesis that the observed differences in bargaining behavior are explained by a higher degree of fairness in resettled communities alone. Moreover, the data reject all model specifications that assume similar fairness in favor of specifications that estimate higher fairness in resettled villages (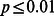 ). In the regional estimations of the model, higher fairness is also estimated for resettled villagers within each area, but the difference in the estimates of resettled and traditional villagers is small and insignificant for all compared model specifications in Mupfurudzi (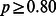 ). In contrast, the homogeneity of resettled and traditional villagers is rejected in favor of models that assumes higher average degrees of fairness and/or rationality in the resettled communities of Mutanda (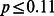 ) and Sengezi (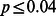 ). The exact p-values depend on the model specifications compared. In Mutanda, for instance, the estimated fairness difference is significant at the 1 and 2 percent levels if rationality is assumed not to vary across resettlement status in the null hypothesis; it is significant at the 11 percent level if rationality is assumed to vary across resettlement status in the null hypothesis. In fact, to avoid sequential testing, for which the test-statistics do not account, it is necessary to depict only one model specification as the null hypothesis.

Throughout, I offered three model choices for the null hypothesis: no fairness and rationality difference; no fairness difference, but rationality difference; and fairness difference, but no rationality difference. If tests of the same alternative model that are based on different null hypotheses point in the same direction, then the *ex ante* decision for the null hypothesis is not necessary to arrive at the same conclusion, and the results are more robust. This is the case for Mupfurudzi and Sengezi. As Mutanda is the smallest subsample with 38 villagers, tests for this area may be less powerful than tests in the larger subsamples of Sengezi with 128 villagers or Mupfurudzi with 68 villagers. Thus, I suggest to interpret the overall test results in Mutanda as evidence for higher fairness among its resettled villagers. The fewer decision errors made by resettled villagers in Sengezi and Mutanda may reflect an increased experience in cooperating with a randomly matched co-villager acquired in the past, which in turn is promoted by the more pronounced fairness preferences.

Against the backdrop of the different regional estimation results, I found one of various differences across resettlement schemes of particular interest. Tribal trust lands and protected villages where the homes to 73, 84 and 91 percent of the settlers in Mupfurudzi, Sengezi and Mutanda, respectively. However, only 10 percent of the resettled villagers in Mupfurudzi lived in tribal trust lands compared to 83 percent in Sengezi and 87 percent in Mutanda (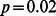 ) [10].

To conclude, the equal or higher degrees of fairness estimated from ultimatum game behavior for resettled villagers represent an equal or stronger interdependence of their well-being. This finding indicates that social cohesion amongst randomly teamed villagers is likely to be present in the new communities to a comparable or higher degree than in traditional communities. As a higher degree of interest in equal performance is less likely to be overruled by self-interest, it gives cause for more stable cooperation, for instance, in public goods provision arguably much needed to successfully manage a new start. The ability to cooperate can be conducive to economics success, regardless of whether it was inherited or brought about by resettlement.

As the new communities are likely to have achieved an equilibrium of their social development almost two decades after resettlement, finding positive degrees of fairness in the resettled villages suggest that the Zimbabwean *willing-buyer and willing-seller* land reform has not eradicated social cohesion, irrespective of whether settlers were more or less fair-minded from the outset. In a complementary analysis of social consequences of the Zimbabwean land reform, *Barr* studied Trust Game behavior in the same resettlement schemes. Assessing data and stylized facts, she suggested that altruistic motivations matter less while motivations relating to a desire to community-built matter more in resettled communities [39]. This conjecture is supported by the estimated fairness attitudes put forward in this study, because fairness concerns, a form of conditional altruism, provide one plausible explanation for cooperativeness. This positive view on the social cohesion in resettlement communities is against the backdrop that, according to *Kinsey*, the early years were a Golden Age for the Zimbabwean land reform program, in which beneficiaries received exceptional levels of supporting services [12].
